# Seismic Performance of Bridge Piers Constructed with PP-ECC at Potential Plastic Hinge Regions

**DOI:** 10.3390/ma13081865

**Published:** 2020-04-16

**Authors:** Yi Jia, Renda Zhao, Fuhai Li, Zhidong Zhou, Yongbao Wang, Yulin Zhan, Xianming Shi

**Affiliations:** 1 Department of Bridge Engineering, Southwest Jiaotong University, Chengdu 610031, China; jiayi201912@163.com (Y.J.); rendazhao@163.com (R.Z.); yulinzhan@home.swjtu.edu.cn (Y.Z.); 2 Department of Civil & Environmental Engineering, Washington State University, Pullman, WA 99164, USA; xianming.shi@wsu.edu; 3 Department of Building Materials, School of Civil Engineering, Southwest Jiaotong University, Chengdu 610031, China; 4 Key Laboratory of High-Speed Railway Engineering, Ministry of Education, Southwest Jiaotong University, Chengdu 610031, China; 5 College of Architecture and Civil Engineering, Taiyuan University of Technology, Taiyuan 030024, China; yonbaowang@163.com

**Keywords:** bridge pier, PP-ECC, seismic performance, pseudo static test, energy dissipation, displacement ductility, hysteretic response

## Abstract

This work presents an experimental investigation on the seismic performance of bridge piers constructed with polypropylene fiber reinforced engineered cementitious composite (PP-ECC) at potential plastic hinge regions. Eight solid square bridge piers are tested under a combination of reversed cyclic lateral loading and constant axial vertical loading. The test variables include the reinforcement stirrup ratio (0 vol.%, 0.46 vol.%, and 0.79 vol.%), axial compression ratio (0.1 and 0.3) and height of the PP-ECC regions (0, 250, and 500 mm). Seismic performance of eight specimens is presented and interpreted, including the failure mode, hysteretic curves, loading–resistance capacity, ductility, stiffness degradation, energy dissipation, and equivalent viscous damping ratio. The material test on the PP-ECC plate specimen suggests that the PP-ECC has obvious strain-hardening behavior and multiple fine-cracking characteristics, with the tensile strength and strain capacity greater than 3.2 MPa and 2.6%, respectively. The PP-ECC material applied at the potential plastic hinge regions notably improves the seismic performance and damage tolerance of bridge piers. The influence of the aforementioned crucial parameters has also been investigated in detail. The axial compression ratio and the height of PP-ECC region have a major influence on the seismic performance of PP-ECC piers. In comparison, the stirrup ratio has a limited effect on the seismic behavior of PP-ECC piers. The experimental findings shed light on the mechanism of the PP-ECC that contributes to the seismic performance of bridge piers and provide some valuable guidance in the seismic design of PP-ECC piers.

## 1. Introduction

Concrete bridges are one type of widely used structures for civil infrastructure system. Concrete bridges require excellent seismic performance to sustain their safety and serviceability after an earthquake exposure. Failures of concrete bridges under seismic loading may lead to thousands of deaths and billions of dollars in repairs and reconstruction (*Analysis of Recent Bridge Failures in the United States* by Kumalasari Wardhana and Fabian C. Hadipriono). During natural earthquake disasters such as the 1994 Northridge (USA), 2008 Wenchuan (China), and 2011 Tohoku (Japan) earthquakes, the collapse of concrete bridges is mainly attributed to insufficient ductility, toughness, and energy dissipation of bridge piers. 

There are two common approaches performed by engineers to enhance the deformation capacity of bridge piers. One method is to increase the amount of reinforcement stirrups and modify the form of stirrups in the plastic hinge region at the bottom of bridge pier. However, also it brings some difficulties in fabricating the steel reinforcement cage and transporting and vibrating concrete during the construction [1]. In addition, this method only increases the ductility of bridge pier to a limited extent [2]. Another method is to incorporate a proper amount of fibers in concrete to improve its ductility and energy absorption. Fiber reinforced concrete (FRC) or fiber reinforced engineered cementitious composites (ECC) have the potential to improve the seismic resistance of bridge pier due to the substantial strain capacity and crack control capability [3,4,5].

Previous studies have demonstrated that ECC or FRCs for beam-column joint connections, columns, beams, and shear walls result in obvious enhancements of the mechanical performance [6,7,8,9]. Zhang et al. [1,10] experimentally investigated the seismic performance of beam–column joint connections constructed with polypropylene fiber reinforced engineered cementitious composite (PP-ECC). They found that PP-ECC was effective in replacing transverse reinforcements and providing sufficient shear strength and dissipating more seismic energy. Zhang et al. [6,11] investigated the effect of shear reinforcement quantity on the shear capacity of PP-ECC beams subjected to four-point bending. The shear force carried by the PP-ECC decreased as the shear reinforcement ratio increased, due to the sliding on the critical crack surface that damaged the PP fibers for crack bridging. Dang et al. [7] conducted some experimental investigation on the seismic behavior of fiber reinforced concrete (FRC) shear walls. They concluded that the proposed FRC shear walls had much better damage tolerance and deformation capacity than those of conventional reinforced concrete (RC) shear walls. Kawashima et al. [3] investigated the effect of polypropylene fiber reinforced cement composite (PP-FRC) on the seismic response of bridge columns subjected to combined axial and bilateral cyclic loadings. They found that strains of tie bars in the PP-FRC column were smaller than those in the conventional RC column. This was due to the larger confinement effect of PP-FRC that originates from the fiber bridging action. PP-FRC is beneficial to allow the structure to remain serviceable even after a strong earthquake. Han et al. [8] found polyvinyl alcohol fiber-reinforced concrete (PVA-FRC) columns exhibited better plastic deformation capacity and damage tolerance as well as better energy dissipation capacity and seismic performance than conventional RC columns. Daniel et al. [12] investigated the effect of steel fiber length on scaled FRC beams subjected to cyclic loadings. As the length of steel fiber increased, crack initiation was delayed at the prepeak stage and the number and length of cracks were reduced. Shin et al. [13] investigated the effectiveness of low-cost FRCs on improving the seismic performance of hollow bridge columns. They found that specimens with higher fiber content achieved larger displacement ductility and greater energy dissipation, while normal concrete columns showed very limited ductility.

PP-ECC features higher tensile strength as well as strain capacity between 3% and 5% under uniaxial tension. The latter is attributable to its unique multicracking mechanism, in which multiple, closely spaced fine cracks form as a result of the bridging action of fibers [14,15]. PP-ECC uses only fine particles in the mix to achieve much denser microstructure [16]. The elimination of coarse aggregate in the PP-ECC mix results in an elastic modulus only half that of ordinary concrete [17,18].

Thus, PP-ECC has been a competent construction material in seismic regions. One application is for bridge piers at the plastic hinge regions to bear large plastic deformation and dissipate more seismic energy [9]. However, few studies have been conducted to investigate the application of PP-ECC in the potential plastic hinge regions at the bottom of bridge piers. The structural performance of bridge piers with partial PP-ECC reinforcement also needs to be evaluated, especially after experiencing a considerable seismic attack.

The aim of this work is to evaluate the effect of PP-ECC on the seismic performance of solid square piers, through the comparative analysis of two normal RC specimens and six PP-ECC specimens. Especially, all the specimens were subjected to combined reversed cyclic lateral displacements and constant axial loading. The reinforcement stirrup ratio, axial compression ratio, and height of the PP-ECC at plastic hinge regions are considered to better understand their influences on the seismic performance of bridge piers, including failure mode, lateral load-displacement hysteretic curves, loading resistance capacity, ductility, stiffness degradation, energy dissipation, and equivalent viscous damping ratio.

## 2. Experimental Program and Setup

### 2.1. Test Specimens and Material Properties

**Figure 1:**
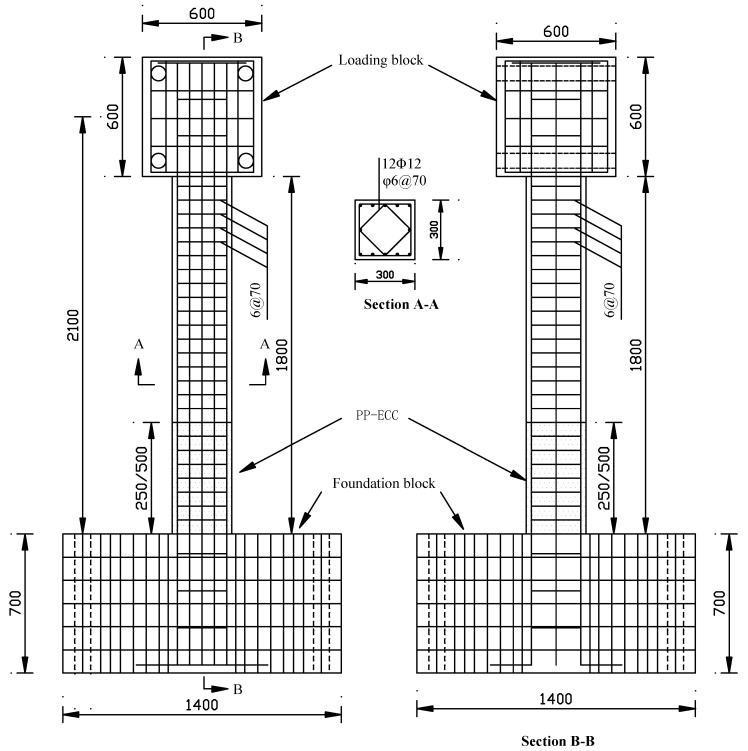
Geometry and reinforcing details of test piers (unit: mm).

In this study, 8 specimens representing cantilever bridge piers constructed with PP-ECC were fabricated and investigated. All the specimens were designed as identical bridge piers with the same dimensions. Figure 1 illustrates the geometry and reinforcement details of bridge piers. The total height of the bridge piers was 3.1 m, and the effective height was 2.1 m (height from the top of the foundation block to the center of the loading block). The shear–span ratio was 7.0 to ensure a flexural failure dominated response. At the bottom of the specimen, a rigid foundation block with dimensions of 1.4 m × 1.4 m × 0.7 m was cast, and the foundation block was fixed to the laboratory floor with four anchor bolts. The cross-section of the piers was 0.3 m × 0.3 m. In order to connect with the MTS actuator, a loading block with dimensions of 0.6 m × 0.6 m × 0.6 m was cast at the top of the specimen.

Table 1 summarizes the design parameters and test variables of eight concrete bridge pier specimens. The main design parameters were the (a) reinforcement stirrup ratio (0 vol.%, 0.46 vol.%, and 0.79 vol.%), (b) axial compression ratio (0.1 and 0.3), and (c) height of the PP-ECC (0, 250, and 500 mm), their values are presented in detail in Table 1. In each bridge pier, twelve steel rebars with a diameter of 12 mm were used as the longitudinal reinforcing bars. They were symmetrically distributed in the cross-section. The resulting longitudinal reinforcing ratio was *ρ*_l_ = 1.51%. The yield strength and ultimate strength of the longitudinal reinforcing bars were 442.5 MPa and 616.8 MPa, respectively. The elastic modulus of the longitudinal reinforcement was 200 GPa, the elongation of longitudinal steel bar after the tensile break was about 24.7%. The steel bar with a diameter of 6 mm was selected for the transverse reinforcement. In order to study the effect of transverse reinforcement ratio at the plastic hinge region on the seismic behavior of specimens, the transverse reinforcement bars were set with the spacing of 500, 140, and 70 mm within the height of 0.5 m measured from the top of the foundation block. The corresponding volume ratios *ρ*_si_ of the transverse reinforcement bars were 0%, 0.46%, and 0.79%, respectively. The transverse reinforcement bars with a spacing of 70 mm was set for the upper part, and the corresponding volume ratio was *ρ*_so_ = 0.79%. The yield strength and ultimate strength of the transverse reinforcement bar was determined as 440.6 MPa and 612.4 MPa, respectively. Elastic modulus of longitudinal reinforcement was 198.7 GPa, the elongation of longitudinal steel bar after tensile break was about 25.3%. A concrete or PP-ECC cover of 20 mm was provided in all the bridge piers.

Table 2 summarizes the physical and mechanical properties of polypropylene fibers. The target compressive strength of the PP-ECC was 30 MPa. The mixture proportion of PP-ECC used in this study was based on a large number of previous mix ratio experiments [1,10]. Ordinary Portland cement, water, fly ash, water reducer, and 2% volume fraction of PP fibers were combined using the mixture proportion listed in Table 3.

### 2.2. Experimental Setup and Loading Protocol

**Figure 2:**
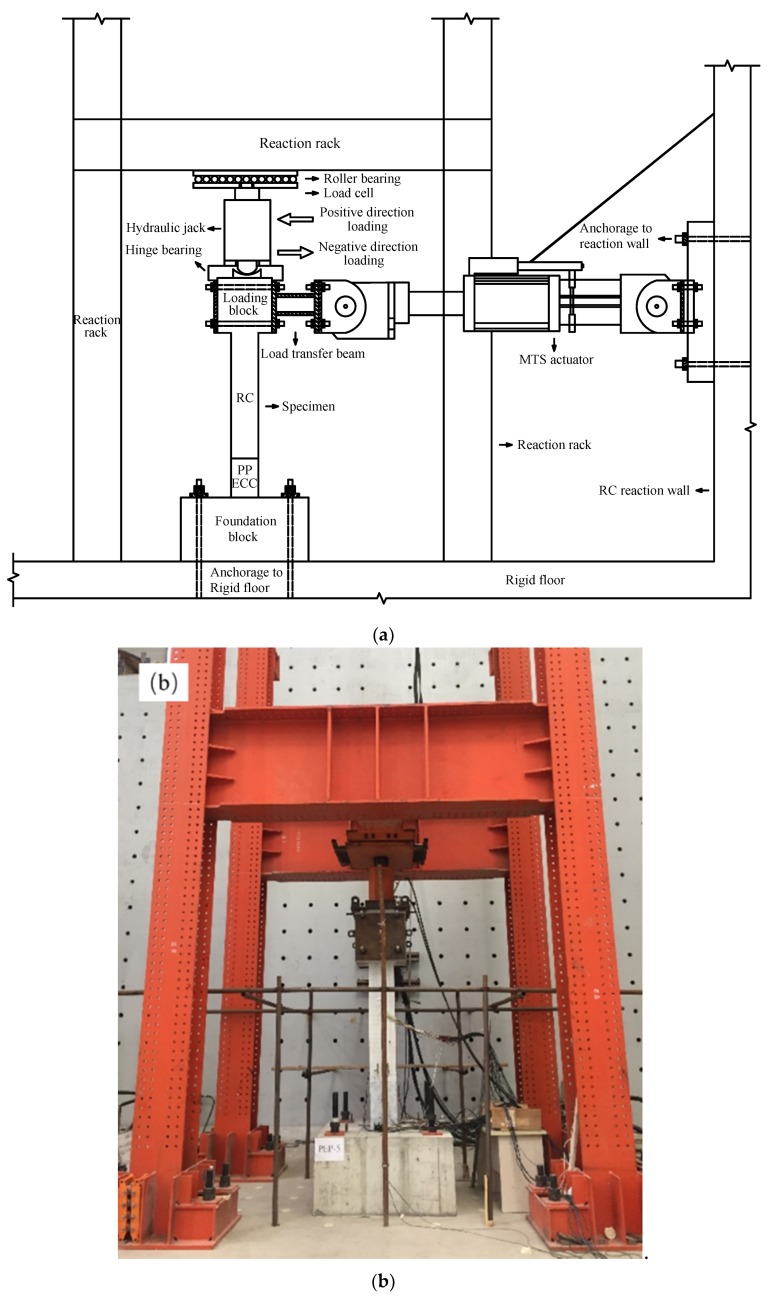
Experimental setup: (**a**) schematic diagram of the test instrumentation and (**b**) photo of the experiment.

In order to investigate the seismic performance of constructed bridge piers, the cyclic loading experiment was conducted at the Southwest Jiaotong University in Chengdu, China. Figure 2 illustrates a schematic diagram of the test instrumentation and photo of the experimental process. All the bridge piers were subjected to constant axial compression force under low cycles of lateral loads [19,20]. To maintain the constant axial load applied on the top of the specimen, a hinge bearing, and a roller bearing were installed at the top of the bridge pier. A 3200 kN hydraulic jack was installed between the hinge bearing and the roller bearing. The low cyclic lateral load was applied to the loading block of the specimen using a 1000 kN MTS actuator. The base of the test piece was anchored on the test bench by four anchor bolts to ensure that the pier specimen did not shift and rotate horizontally during the test. Positive and negative loading directions are shown in Figure 2.

**Figure 3:**
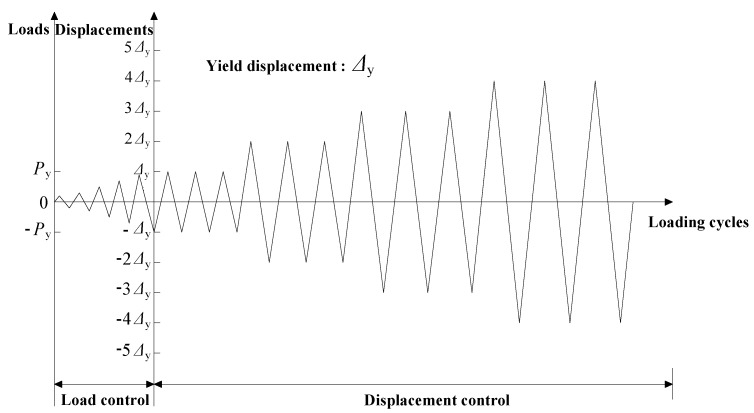
Loading protocol.

During the test, the axial loading was first applied to the specimen using a hydraulic jack and then kept constant. Then, the lateral cyclic load was applied to the specimen by the MTS actuator. Figure 3 depicts the lateral loading protocol used in the test. As illustrated in the figure, the loading protocol involved two load steps [21]. In the first step, the displacement levels were applied under a load-controlled mode. They were reversed only one time per each level, the increment of lateral cycle load was 5 kN. After the longitudinal reinforcement at the bottom of the pier yielded, the loading protocol of specimens was then changed into the displacement-controlled mode, and each displacement level was repeated three times. The increment of the displacement was denoted as the yield displacement *Δ*_y_ of the specimen for the displacement-controlled cyclic loading test, where the excited displacements were *Δ*_y_, 2*Δ*_y_, 3*Δ*_y_, etc. *Δ*_y_ refers to the lateral displacement causing the yield of the longitudinal reinforcement. Finally, the experiments were terminated when the lateral load of the specimen dropped below 85% of its peak value during the cyclic loading test.

## 3. Material Properties of Concretes

### 3.1. Compressive Test

Compression tests were carried out on cubic specimens to study the compressive characteristics of PP-ECC and RC. Three PP-ECC and RC standard cubic specimens with a length of 150 mm were prepared. After 28 days of standard curing in a room temperature of 20 ± 5 °C and relative humidity of 95%, the compressive strength of PP-ECC and RC was determined to be 28.6 ± 0.8 and 27.2 ± 0.6 MPa, respectively. The strain corresponding at the peak stress of PP-ECC and RC were 0.40% and 0.19%, respectively. It is noteworthy that the elastic moduli of PP-ECC and RC were quite different. The elastic moduli of PP-ECC and RC were 15.7 ± 0.22 GPa and 28.5 ± 0.26 GPa, respectively. This significant difference of elastic modulus may be attributed to the fundamental difference in the mix design of PP-ECC and RC, as the elimination of coarse aggregate in ECC is known to result in lower elastic modulus [1,10].

**Figure 4:**
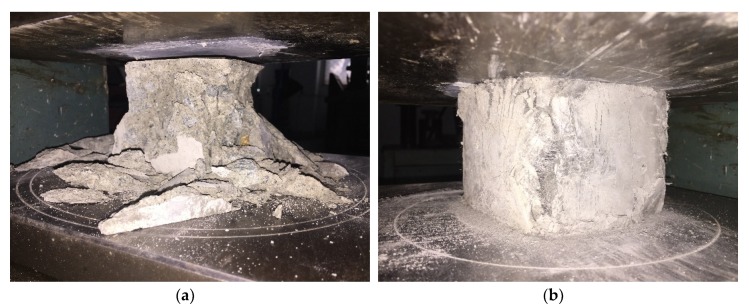
Compressive failure modes of cubic specimen. (**a**) Reinforced concrete (RC) cubic specimen. (**b**) PP-ECC cubic specimen.

The compressive failure modes of RC and PP-ECC cubic specimens are illustrated in Figure 4. As shown in Figure 4, the typical compressive response of the RC cubic specimen was brittle failure with a large area of spalling and splitting. However, the PP-ECC cubic specimens exhibited more excellent ductility than the RC ones. When the PP-ECC cubic specimens failed, they still maintained good integrity. Numerous vertical fine cracks were formed, and the bearing capacity decreased slowly.

### 3.2. Tensile Test of PP-ECC

**Figure 5:**
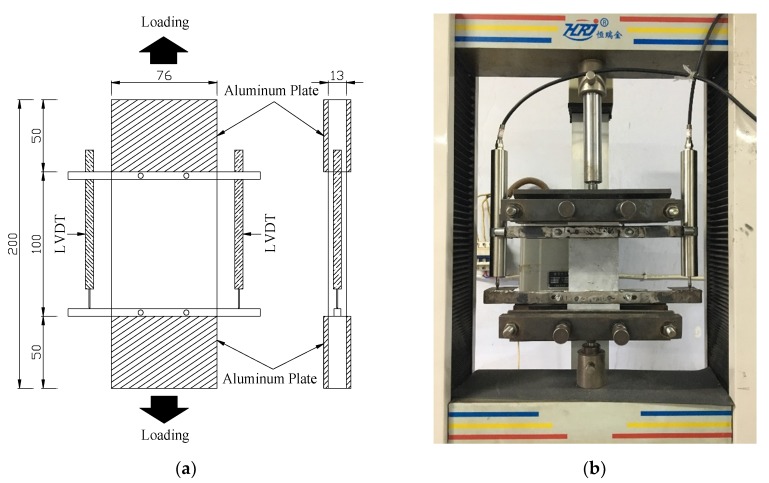
PP-ECC specimen configuration for uniaxial tensile tests (unit: mm). (**a**) Dimensions of specimen and (**b**) test setup.

In order to investigate the most important tensile properties of PP-ECC, the uniaxial tensile tests with rectangular plate specimens were carried out, as shown in Figure 5. The designed dimension of specimens was similar to those developed by the Tokyo Institute of Technology research team [1]. The cross section of the PP-ECC plate was 76 mm × 13 mm. The length of the specimen was 200 mm, including 50 mm connecting length at each end of the PP-ECC plate specimen for gripping fixture. The 100 mm in the middle of the PP-ECC plate specimen was used as the gage length. To capture the axial tensile deformation of the PP-ECC plate specimen, two linear variable differential transformers (LVDTs) were installed in parallel to the loading direction at both sides of the specimen. The uniaxial tensile tests were performed under a constant displacement rate of 0.08 mm/min. Tension tests were stopped when a crack of the specimen appeared localized propagation failure.

**Figure 6:**
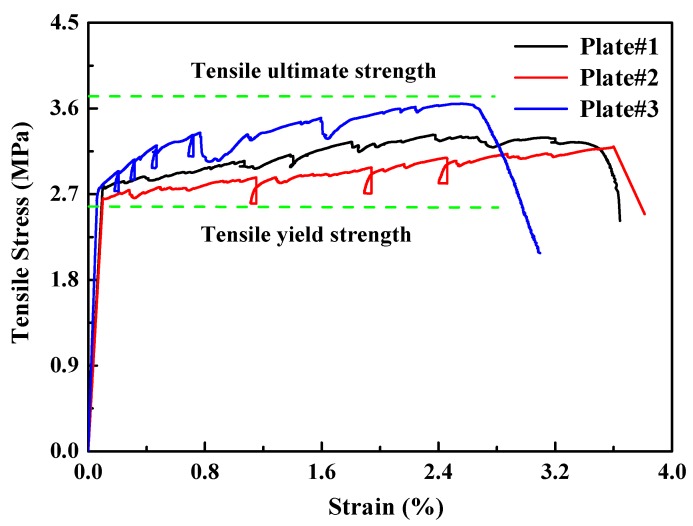
Uniaxial tensile stress–strain responses of PP-ECC.

**Figure 7:**
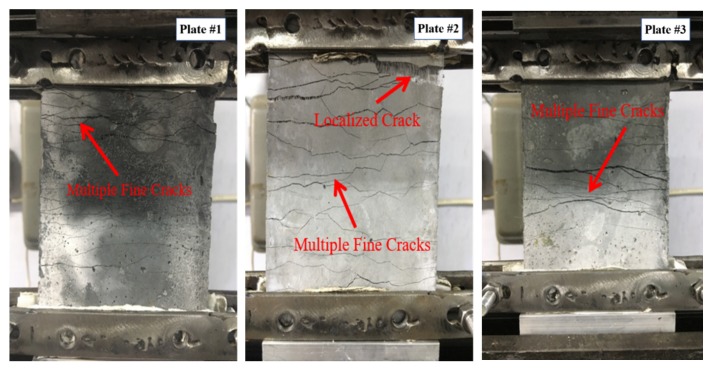
Tensile failure modes of PP-ECC.

Figure 6 shows typical uniaxial tensile stress–strain curves of the three PP-ECC plates. All specimens exhibited apparent strain-hardening characteristics. The characteristic strain-hardening behavior after first cracking was accompanied by multiple fine-cracks and the tensile failure modes of PP-ECC were shown in Figure 7. The tensile loading of the specimen was known as the yield strength of PP-ECC when the first microcrack appears. The tensile yield strength of the PP-ECC was approximately 2.7 MPa. The tensile ultimate strength was defined as the maximum stress based on the uniaxial tensile stress–strain responses. The tensile ultimate strain was defined as when a localized cracking gradually widened, and the test loading began to decrease notably. The uniaxial tensile ultimate strength and strain capacity were in the ranges of 3.2–3.6 MPa and 2.6–3.6%, respectively.

## 4. Results and Discussion

### 4.1. General Test Observations and Failure Modes of Piers

**Figure 8:**
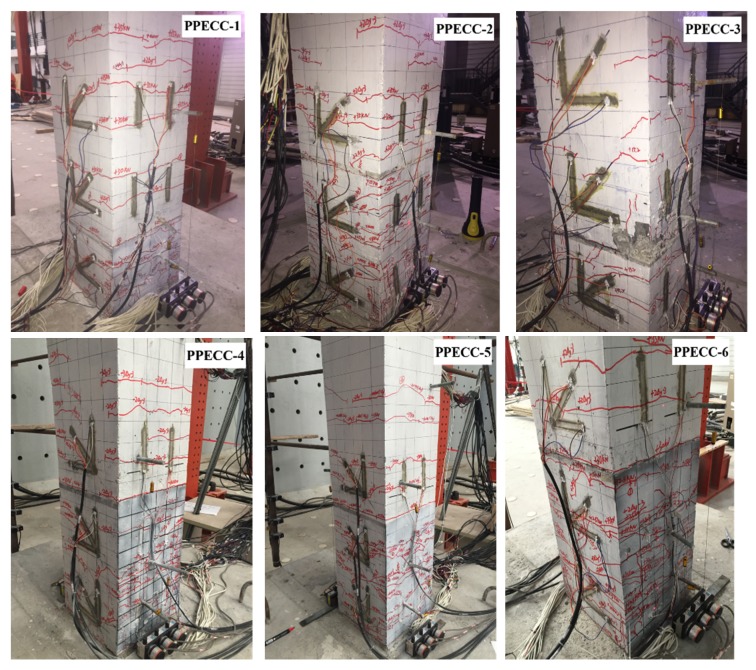

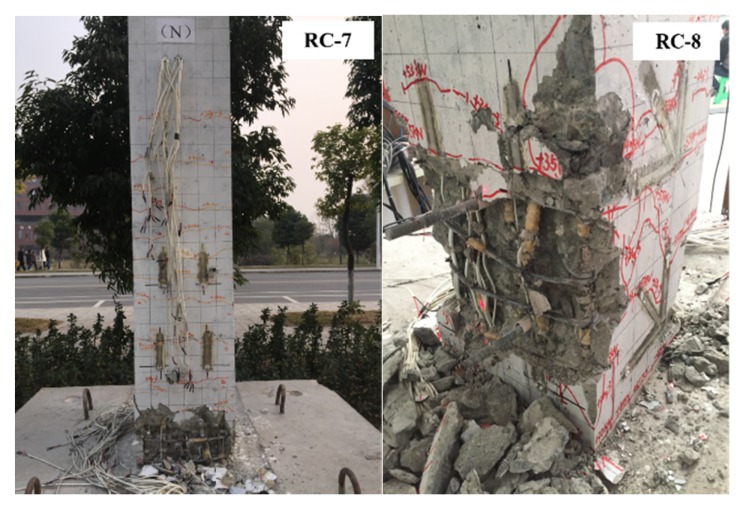
Crack patterns and failure modes of specimens.

Figure 8 illustrates the cracking patterns and typical failure modes of eight pier specimens observed after testing. The failure processes of the PP-ECC and RC specimens are carefully monitored and recorded during the experiment, the details are described as follows.

For the PP-ECC specimens, six specimens (PPECC1–PPECC6) exhibited similar failure process and modes. The scenario of specimen PPECC6 was discussed here. The first crack usually occurred at approximately 13 cm above the bottom of the pier, when the loading was 10 kN. As the test load increased, several micro horizontal cracks and oblique shear cracks were formed in the PP-ECC region at the bottom of the pier. The spacing between adjacent cracks was about 4–6 cm. Before the longitudinal steel bars yields, the cracks were observed to be completely closed during unloading, and the residual deformation of the specimen was extremely small. The loading curve of the test coincides with the unloading curve into a straight line, indicating that the specimen was in the elastic stage. Numerous horizontal cracks were formed in the PP-ECC areas of the pier when the longitudinal reinforcement yielded, while the horizontal yield load was 37 kN. During the test, fibers in the PP-ECC were tensile failure associated with a “hissing” sound. When the lateral displacement reached 80 mm, the lateral loading reached 51.3 kN, several horizontal cracks occurred in the non-PP-ECC region of the specimen, and the spacing between the adjacent cracks was about 15 cm. When the peak loading was reached, the width of existing cracks increased and fewer new cracks occurred during the load dropping. A large number of vertical cracks occurred within the range of 15 cm from the bottom of the pier. In the range of 15–20 cm from the bottom of the pier, the local PP-ECC had bulged outwards under pressure, but the protective layer of PP-ECC was not peeled off and crashed over the test period. When loaded to 100 mm, the bearing capacity of the specimen had dropped to less than 85% of the peak loading, the internal longitudinal steel bars of the specimen buckled significantly, and then the test was stopped. Due to the crack bridging and deflecting effects of fibers in the PP-ECC pier, the PP ECC material effectively controlled the propagation and development of cracks. As such, the specimen preserved its good integrity even after failure, which improved the damage tolerance of such a pier.

For the RC specimens, both RC7 and RC8 specimens exhibited similar failure processes and modes. They failed in a flexural manner. The scenario of specimen RC8 was discussed here. The specimen RC8 experienced four typical failure stages during the test, such as cracking of concrete, yield of steel bar, spalling of concrete cover, and buckling of longitudinal reinforcement. In the initial stage of the test, there were no cracks on the surfaces of specimen RC8 because the deformation of the piers was elastically forced. The first microcrack occurred in the plastic hinge region when the test loading was 20 kN. As the lateral loading kept increasing, the number and width of cracks on the surface of the specimen increased. The spacing between adjacent cracks was 6 cm, and the cracks propagated to both sides. The internal longitudinal reinforcement of specimen yields at a test loading of 55 kN, and the corresponding lateral displacement was 20 mm. Displacement-controlled cyclic loading was then applied. Crack width and length increase with the increasing lateral displacements, more new horizontal cracks appeared in the middle and lower part of pier, and the spacing between adjacent cracks was about 10 cm. As the displacement continued to increase, fewer new cracks formed. However, the previously formed cracks became wider and longer gradually, and four main cracks formed in the plastic hinge region at the lateral displacement of 40 mm. When the displacement reached 60 mm, some concrete cover was crushed and peeled off. At the same time, the longitudinal steel bars and stirrups were exposed and a distinct plastic hinge was generated at the end of the pier as well. At the lateral displacement of 80 mm, the maximum loading was achieved in this test, majority of concrete cover at the bottom of the pier were crushed and peeled off. The longitudinal reinforcements were buckling due to high stress, and the hoops were pulled outward. The bearing capacity of the specimen reduced to less than 85% of the peak loading, indicated that the specimen RC8 was destroyed and then the test terminates. Although there were some differences between reinforced concrete pier and PP-ECC pier, they all belonged to the bending failure mode.

### 4.2. Lateral Load-Displacement Hysteretic Curves

**Figure 9:**
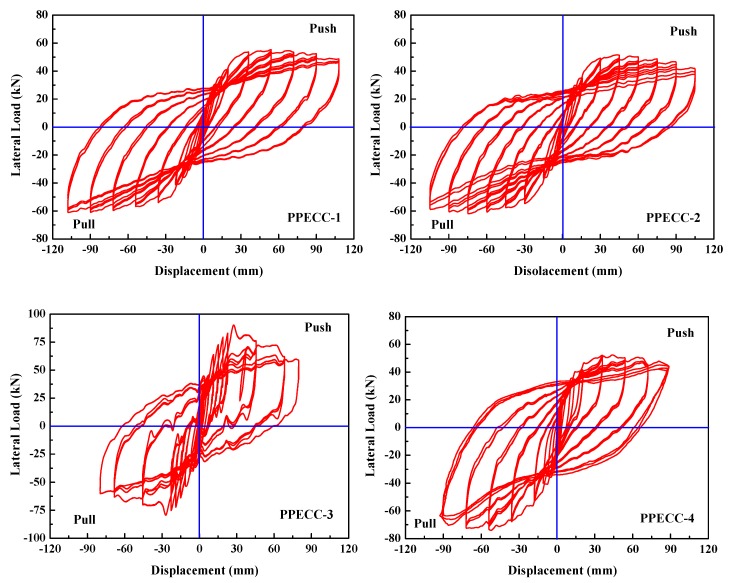

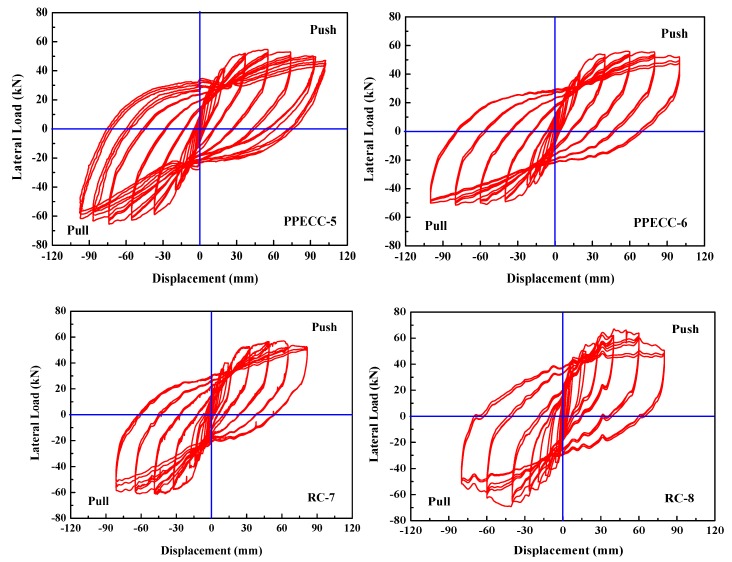
Lateral load–displacement hysteretic curves of specimens.

Figure 9 shows the lateral load–displacement hysteretic curves for all the specimens obtained from the cyclic loading tests. It represents the relationship between the lateral load and displacement at the top of the specimens. At the early stage, before the yielding of the longitudinal reinforcement of the specimen, the stiffness of loading and unloading almost did not change and there was small residual deformation after unloading. The shape of hysteresis curves of all specimens was very similar, and the area of hysteresis loop increased with the increase of lateral load. Before the peak loading, the stiffness of each specimen did not degrade obviously, the lateral load increased with the increase of measured displacement. After the peak loading, the load–bearing capacity of the specimens decreased gradually with the increase of lateral displacement, the stiffness also exhibited an obvious degradation phenomenon and the residual deformation increased gradually. The shape of the hysteresis loop was fusiform and had a slight “pinch” effect. PPECC1–PPECC6 specimens had a relatively larger area of hysteretic curves, compared to RC7 and RC8 specimens. The reductions of strength attenuation and stiffness were relatively slower after the peak loading indicating that the seismic resistance of the PP-ECC piers was better than that of ordinary reinforced concrete (RC) piers. It could be contributed to the excellent ductility and energy absorption in PP-ECC piers associated with the fiber bridging effect.

The test variables in the study had different influences on the hysteretic curve of PP-ECC piers. By comparing with PPECC-2, PPECC-5, and PPECC-6, one could conclude that the hysteretic curves became larger and the displacement ductility and energy dissipation capacity increased with the increase of the stirrup ratio in the plastic hinge region. In short, the PP-ECC piers exhibited better seismic resistance when the stirrup ratio was relatively high. This is consistent with the effect of the stirrup ratio on the seismic behavior of ordinary reinforced concrete pier [9].

The influence of the axial compression ratio on the hysteretic curves of PP-ECC piers was obvious. By comparing PPECC-1–PPECC-4, the area of hysteretic curves of PPECC-1 and PPECC-2 were larger than those of PPECC-3 and PPECC-4. It indicates that the smaller the axial compression ratio, the better the deformation and energy dissipation. The PP-ECC pier with high axial compression ratios accelerated the bearing capacity and stiffness degradation after peak loading. This phenomenon was more obviously shown in the PPECC-3 specimen since its axial compression ratio was 0.3. It was implied that decreasing the axial compression ratio properly could improve the seismic performance of PP-ECC piers.

The height of the PP-ECC region had a very limited influence on the hysteretic curves of PP-ECC piers. When the height of the PP-ECC region increased from 250 to 500 mm, the area of the hysteretic curve of PP-ECC specimens increased slightly. Both the strength attenuation and stiffness degradation were improved to some extent. It seemed that the PP-ECC piers with a higher PP-ECC region would have better seismic behavior due to superior ductility properties of PP-ECC materials.

### 4.3. Lateral Resistance Capacity and Ductility

**Figure 10:**
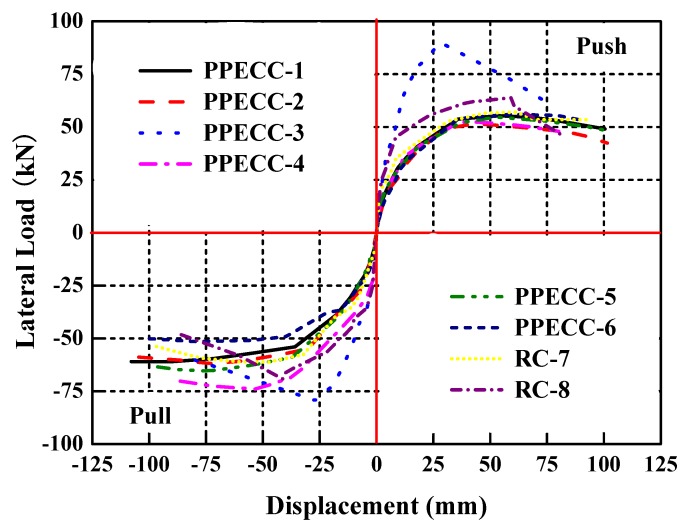
Envelope curves of PP-ECC and RC piers.

**Figure 11:**
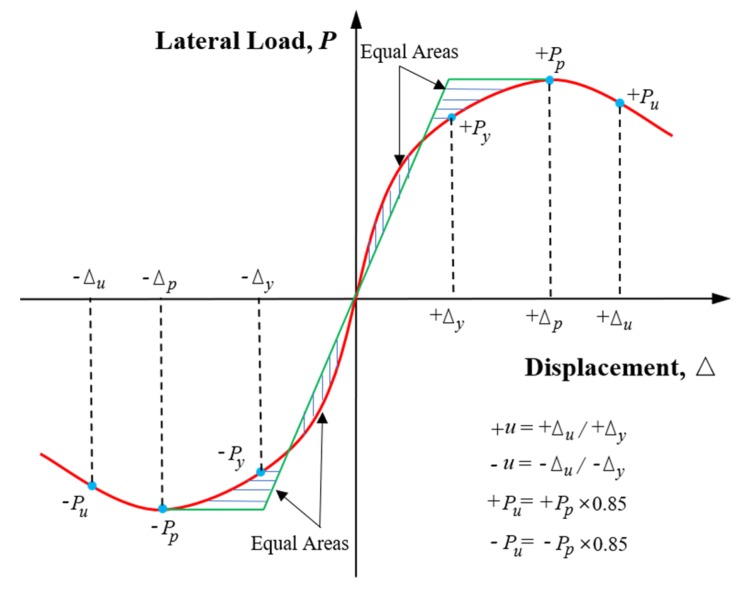
Definition of characteristic points of envelope curves [24].

The envelope curve reflected the relationship between the peak load and the corresponding displacement of the specimens in each loading stage. Figure 10 shows the lateral load–displacement envelope curves of all the specimens obtained from the hysteretic curves. Compared to the specimens RC-7 and RC-8, there were no discernible reductions in loading-resistant capacity and stiffness of all PP-ECC specimens before failure except for the specimen PPECC-3. The obvious deterioration of strength and stiffness of the specimen PPECC-3 could be contributed to the relatively higher axial compression ratio and lower PP-ECC region. The characteristic loads and displacements in the envelope curves are defined and illustrated in Figure 11 [22]. As shown in Figure 11, the yield load and the corresponding displacement of each specimen are obtained using the energy method proposed by Mahin and Bettero [23,24]. The ultimate load and the corresponding displacement of each specimen were determined according to the common definition of loading–resistance reducing to 85% of the peak load. The coefficient of displacement ductility, which is defined as the ratio of ultimate displacement to yield displacement, reflects the inelastic deformation capacity of the specimen when the bearing capacity did not decrease significantly. The envelope curves obtained from the experiments were almost asymmetrical, the values in the positive and negative directions were investigated and summarized in Table 4. The average values of the positive and negative directions of each parameter were calculated and summarized in the table as well.

The axial compression ratio obviously affected the characteristic loads and displacement ductility of the PP-ECC and RC specimens. When the axial compression ratio increased from 0.1 to 0.3, for the PPECC-1 and PPECC-3 specimens with a PP-ECC height of 250 mm in the plastic hinge region, the yield loads, peak loads, and ultimate loads show reductions of 97.8%, 45.7%, and 45.7%, respectively, while the coefficient of displacement ductility decreased by 51.7%. For the PPECC-2 and PPECC-4 specimens with a PP-ECC height of 500 mm in the plastic hinge region, the yield loads, peak loads and ultimate loads were increased by 13.2%, 11.3%, and 11.4%, respectively, while the coefficient of displacement ductility decreased by 30.1%. For the RC-7 and RC-8 specimens, the yield loads, peak loads, and ultimate loads increased by 40.5%, 12.5%, and 12.5%, respectively, while the coefficient of displacement ductility decreased by 47.2%. These observations suggest that bearing capacity of the PP-ECC and RC piers increased sizably with an increasing axial compression ratio, but the displacement ductility coefficient shows the opposite trend. Therefore, the axial compression ratio was not recommended to be too high for seismic designs of PP-ECC piers.

The characteristic loads and the displacement ductility of the PP-ECC piers (i.e., PPECC-2, PPECC-5, and PPECC-6) were obviously influenced by the stirrup ratio. From Table 4, it was observed that the yield loads, peak loads, and ultimate loads increased first and then decreased with the increase of the stirrup ratio. Therefore, the PPECC-5 specimen with a stirrup ratio of 0.46% in the plastic hinge regions had the highest load bearing capacity, compared to the other two specimens. The coefficient of displacement ductility increased gradually with an increasing of stirrup ratio. Compared with PPECC-6 specimen without stirrups, the coefficients of displacement ductility of PPECC-5 and PPECC-2 specimens were higher by 6.7% and 33.9%, respectively. It indicated that a high stirrup ratio in the PP-ECC specimen was beneficial to the displacement ductility.

The height of PP-ECC in the plastic hinge regions had obvious effects on the characteristic loads and displacement ductility of the specimens (i.e., RC-8, PPECC-3, and PPECC-4). Compared to RC-8 specimen, the yield loads, peak loads, and ultimate loads of PPECC-3 specimen were higher by 40.7%, 27.3%, and 27.4%, respectively. However, the yield loads, peak loads and ultimate loads of the PPECC-4 specimen were respectively 28.3%, 5.1%, and 5.1% lower. The PPECC-3 specimen was the highest bearing capacity among these three scenarios. As the height of the PP-ECC region increased, the coefficient of displacement ductility gradually increased. Compared to the RC-8 specimen, the coefficients of displacement ductility of PPECC-3 and PPECC-4 specimens were enhanced by 9.4% and 78.1%, respectively. It was concluded that the increasing height of the PP-ECC region was favorable to the ductility property for PP-ECC piers, while unfavorable to the bearing capacity.

### 4.4. Stiffness Degradation 

Stiffness degradation is one of the most important indices to characterize the seismic performance of PP-ECC members and RC structures. In this study, secant stiffness (*K*_i_) obtained from the experimental results was used to characterize the stiffness degradation of specimens under cyclic loading. Considering the asymmetry of the lateral load–displacement hysteretic curves, *K*_i_ was calculated in the positive and negative direction of each hysteretic curve, which is defined as follows [7]:

(1) $$K_{i}=\frac{\left|+P_{i}\right|+\left|-P_{i}\right|}{\left|+\Delta_{i}\right|+\left|-\Delta_{i}\right|}$$

where *P*_i_ is the peak load at the ith displacement level; *Δ*_i_ is the displacement corresponding to the *P*_i_; and the superscripts “+” and “−” represent the positive and negative direction, respectively.

**Figure 12:**
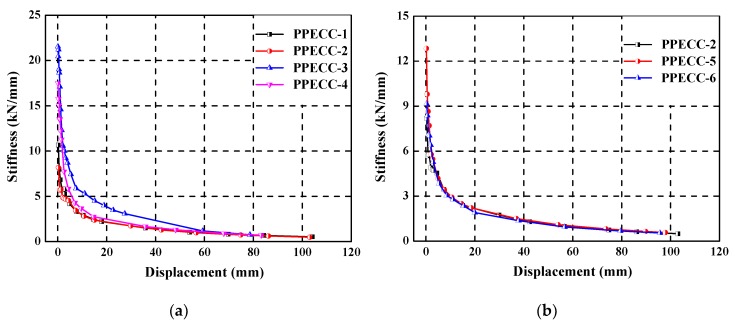

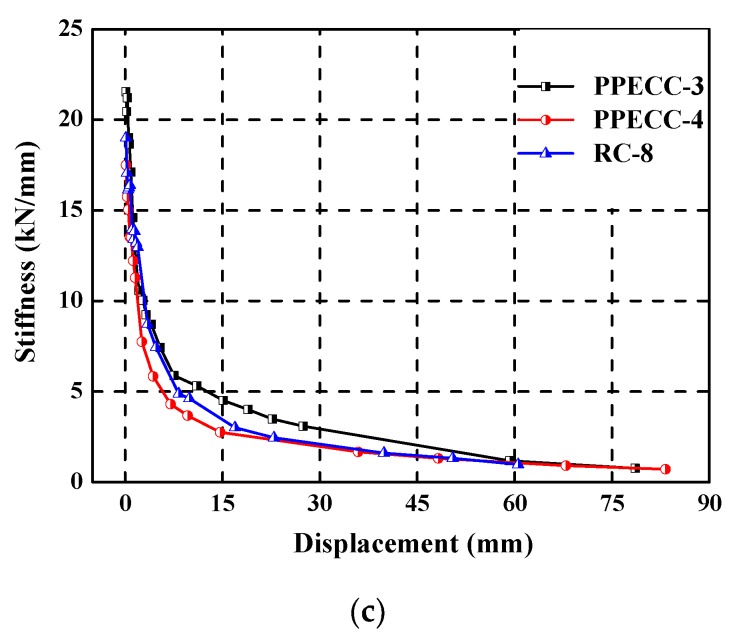
Stiffness degradation of specimens: the influence of (**a**) axial compression ratio, (**b**) stirrup ratio, and (**c**) the height of the PP-ECC region.

Figure 12 illustrates the experimental values of *K*_i_ versus the lateral displacement for all specimens. The initial stiffness of all specimens is relatively high before the specimen cracks. The secant stiffness of all specimens decreased gradually as the lateral displacement increased. Before the yielding of specimens, the secant stiffness of most specimens degenerated very slowly. After that, the stiffness degradation of all the specimens was limited. Beyond that point, the secant stiffness of all specimens decreased more and more smoothly, and the stiffness did not change too much before the failure of piers.

Figure 12a illustrates the influence of the axial compression ratio on the stiffness degradation of the PP-ECC specimens. The higher the axial compression ratio, the greater the initial stiffness of the specimen it exhibited. After cracks occurred in the PP-ECC specimens, the increasing rate of stiffness degradation was also higher. In addition, a larger increasing rate of stiffness degradation was shown in the specimen with a relatively higher axial compression ratio. The axial compression ratio exhibited a substantial effect on the stiffness degradation of PP-ECC piers. Figure 12b depicts that the initial stiffness of PP-ECC specimens was also influenced by the stirrup ratio. The higher the stirrup ratio, the smaller the initial stiffness. However, after cracks appeared, the relation curves between stiffness and displacement of three PP-ECC specimens were almost coincidental, indicating that the stirrup ratio had an insignificant effect on the rate of stiffness degradation. It is evident from Figure 12c that the initial stiffness values were similar for the three piers with different height of the PP-ECC region. The secant stiffnesses of the piers with a high height of the PP-ECC region were smaller than those of the piers with a low height of the PP-ECC region after cracks appeared in the specimens.

### 4.5. Energy Dissipation

**Figure 13:**
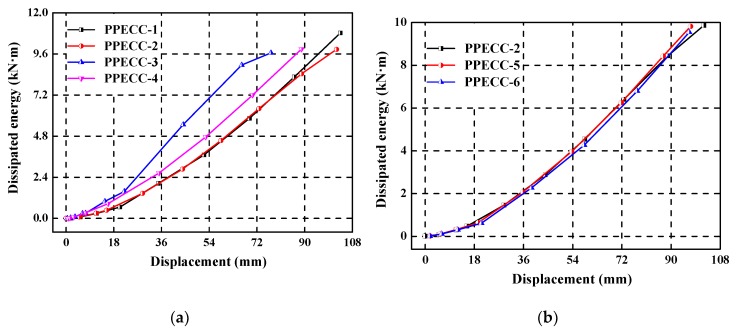

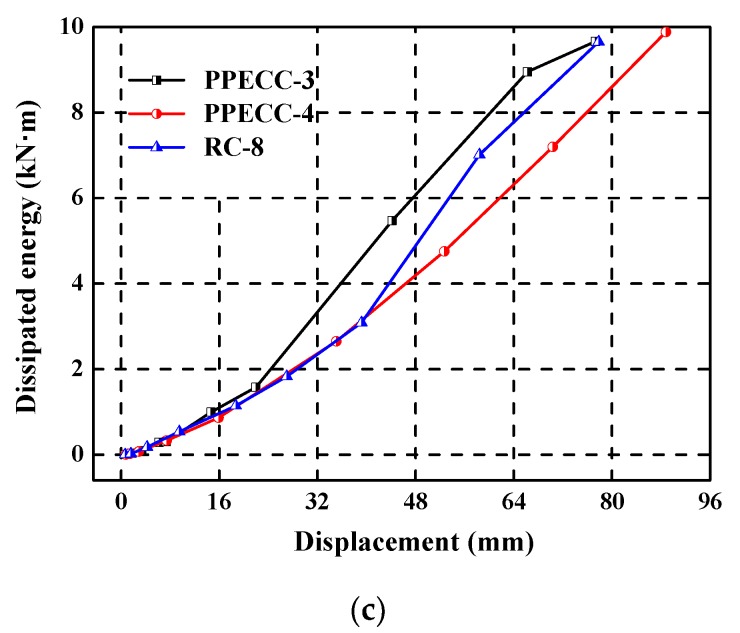
Dissipated energy versus the displacement, the influence of the (**a**) axial compression ratio, (**b**) stirrup ratio, and (**c**) the height of the PP-ECC region.

The energy dissipation capacity is an important character for the seismic performance of PP-ECC and RC structures, it reflects the ability of energy absorption under seismic loading. The area enclosed by the lateral load–displacement hysteretic curves is defined as the energy dissipation capacity of the structures [25]. In the present study, the relationship of energy dissipation versus the lateral displacement of all piers is comparatively plotted in Figure 13.

Figure 13a shows the influence of the axial compression ratio on the energy dissipation of PP-ECC specimens. The dissipated energy increased considerably as the axial compression ratio increases under the same displacement level, indicating that the axial compression ratio had a notable effect on the energy dissipation of PP-ECC piers. Figure 13b illustrates the influence of stirrup ratio on the energy dissipation. There is little difference in the energy dissipation capacity shown among the three specimens with different stirrup ratio (i.e., PPECC-2, PPECC-5, and PPECC-6). It implies that the stirrup ratio had no obvious effect on the energy dissipation of PP-ECC specimens. Figure 13c shows the influence of the height of the PP-ECC region on the energy dissipation. The dissipated energy of the three specimens was almost the same before the reinforcement yields. When the lateral displacement exceeded 40 mm, the dissipated energy capacity decreased first and then increased as the height of PP-ECC region increased under the same displacement level. Therefore, the height of the PP-ECC region should be controlled strictly in the seismic design of bridge piers.

### 4.6. Equivalent Viscous Damping Ratio

Another interesting approach to deal with the energy dissipation is through the evaluation of the equivalent viscous damping ratio *ξ*_eq_. The equivalent viscous damping ratio can be obtained from the test data of the lateral load–displacement hysteretic curves. The definition of equivalent viscous damping ratio in Clough and Penzien [26] was adopted in this study, which is expressed as follows:

(2) $$\xi_{\mathrm{eq}}=\frac{1}{2\pi }\cdot \frac{E_{D}}{E_{S}}$$

**Figure 14:**
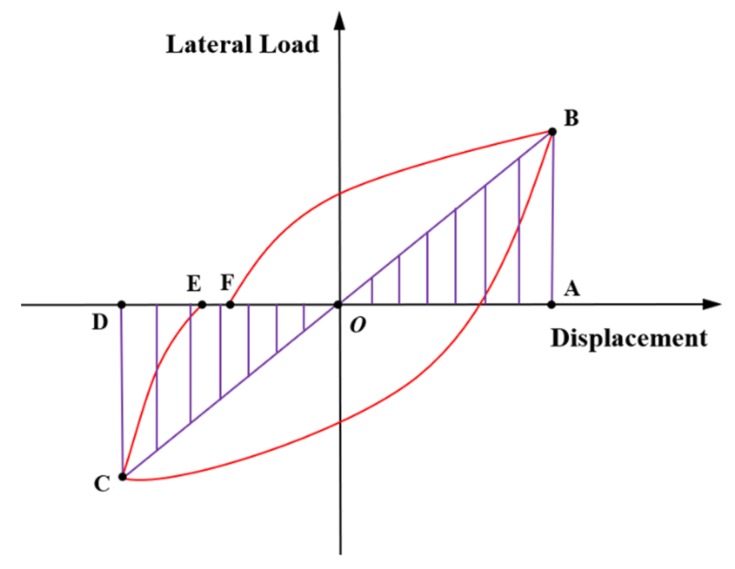
Schematic diagram of the elastic and dissipated energies [27].

The schematic diagram of the elastic and dissipated energies is illustrated in Figure 14 [27], where *E*_D_ is the area of one hysteretic curve (*S*_BCEF_) represents the dissipated energy. *E*_S_ is determined by the sum of the two triangular areas (*S*_ΔOAB_ and *S*_ΔOCD_) represents the elastic energy.

**Figure 15:**
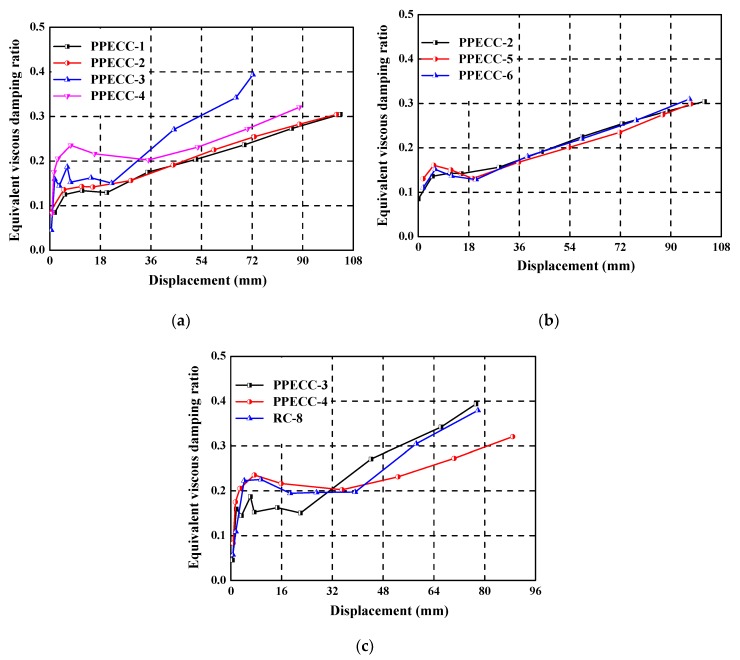
Equivalent viscous damping ratio, the influence of the (**a**) axial compression ratio, (**b**) stirrup ratio, and (**c**) the height of the PP-ECC region.

Figure 15 shows the equivalent viscous damping ratio versus the lateral displacement for PP-ECC and RC piers. It can be seen that the equivalent viscous damping ratio increased with the increase of lateral displacement in the overall trend for all the specimens. The influence of the axial compression ratio on the equivalent viscous damping ratio is shown in Figure 15a. It can be seen that the equivalent viscous damping ratio of the specimens with a high axial compression ratio (i.e., PPECC-3 and PPECC-4) was larger than those of the specimens with a lower axial compression ratio (i.e., PPECC-1 and PPECC-2) under the same displacement level. It implies that increasing the axial compression ratio could improve the energy dissipation capacity of PP-ECC piers. Figure 15b shows the influence of the stirrup ratio on the equivalent viscous damping ratio of the three specimens. Specimens with different stirrup ratios had almost the same equivalent viscous damping ratios at a given displacement amplitude. It seems that the stirrup ratio had limited influence on the equivalent viscous damping ratio of PP-ECC piers. Figure 15c shows the influence of the height of the PP-ECC region on the equivalent viscous damping ratio. It can be seen that the equivalent viscous damping ratios of PPECC-4 and RC-8 specimens were greater than the PPECC-3 specimen under the same displacement level prior to 32 mm lateral displacement. However, beyond 32 mm lateral displacement, the equivalent viscous damping ratios of PPECC-3 and RC-8 specimens were larger than the PPECC-4 specimen at any given displacement level. It revealed that the equivalent viscous damping ratio of the PP-ECC pier was unfavorably affected by the height of the PP-ECC region.

## 5. Conclusions

In this study, a series of experimental investigations were conducted on the solid square reinforced concrete bridge piers subjected to earthquake excitation, with and without PP-ECC at the potential plastic hinge regions. Several crucial parameters were investigated, including the stirrups ratio, axial compression ratio, and the height of PP-ECC regions. Meanwhile, analysis was also performed on the failure modes, lateral load–displacement hysteretic curves, lateral resistance capacity, ductility, stiffness degradation, energy dissipation, and equivalent viscous damping ratio of bridge piers. Based on the experimental results, the following conclusions could be drawn:

(1) The PP-ECC plate specimen clearly exhibited strain-hardening behavior and multiple steady fine-cracking characteristics under the uniaxial tensile test. The tensile strength and strain capacity of the PP-ECC plate were greater than 3.2 MPa and 2.6%, respectively.
(2) The seismic performance of the PP-ECC specimens, including the development and control of cracks, the resistance capacity, ductility property, stiffness degradation, and energy dissipation, were much better than those the reinforced concrete (RC) specimen. Therefore, constructed with PP-ECC at potential plastic hinge regions could effectively improve the seismic behavior and damage tolerance of bridge piers.
(3) The bearing capacity of PP-ECC and RC specimens increased with the increase of the axial compression ratio. Additionally, the ductility coefficient decreased with the increasing axial compression ratio. The PP-ECC piers with a high axial compression ratio accelerated the bearing capacity and stiffness degradation after peak loading. The energy dissipation and equivalent viscous damping ratio of PP-ECC increased considerably as the axial compression ratio increased under the same displacement level.
(4) The displacement ductility of PP-ECC piers was improved with an increase in the stirrup ratio. The stirrup ratio had obvious influence on the initial stiffness of PP-ECC piers but had limited effect on the rate of stiffness degradation after peak loading. It had a negligible effect on the energy dissipation and equivalent viscous damping ratio of PP-ECC piers.
(5) Increasing the height of PP-ECC was favorable to the ductility enhancement, but unfavorable to the bearing capacity and equivalent viscous damping ratio. Therefore, the height of the PP-ECC region should be controlled strictly in the seismic design of PP-ECC piers.

## Figures and Tables

**Table 1:** Design parameters of specimens.

Specimen	*H* (mm)	*λ*	*ρ*_l_ (%)	*ρ*_so_ (%)	*ρ*_si_ (%)	*h* (mm)	*n*
PPECC-1	2100	7.0	1.51	0.79	0.79	250	0.1
PPECC-2	2100	7.0	1.51	0.79	0.79	500	0.1
PPECC-3	2100	7.0	1.51	0.79	0.79	250	0.3
PPECC-4	2100	7.0	1.51	0.79	0.79	500	0.3
PPECC-5	2100	7.0	1.51	0.79	0.46	500	0.1
PPECC-6	2100	7.0	1.51	0.79	0	500	0.1
RC-7	2100	7.0	1.51	0.79	0.79	0	0.1
RC-8	2100	7.0	1.51	0.79	0.79	0	0.3

Notes: *H*: the height of pier; λ: shear span ratio; *ρ*_l_: longitudinal reinforcing ratio; *ρ*_so_: volume ratios of the transverse reinforcement bars of outside the polypropylene fiber reinforced engineered cementitious composite (PP-ECC) region; *ρ*_si_: volume ratios of the transverse reinforcement bars of PP-ECC region; *h*: the height of PP-ECC region; *n*: axial compression ratio.

**Table 2:** Properties of polypropylene fibers.

Fiber	Diameter (μm)	Length (mm)	Density (kg/m^3^)	Stretch Rate (%)	Elastic Modulus (GPa)	Tensile Strength (MPa)
polypropylene	20	12	0.91	15	5	480

**Table 3:** Mixture proportion of PP-ECC.

Cement (kg/m^3^)	Fly Ash (kg/m^3^)	Water (kg/m^3^)	PP Fiber (kg/m^3^)	Water Reducer (kg/m^3^)	W/B (%)	FA/B (%)
820	442	505	18.2	8.834	0.4	0.35

Notes: W: water; B: binder (cement and fly ash); FA: fly ash.

**Table 4:** Capacity and ductility of the specimens.

Specimen	+*P*_y_/−*P*_y_ (*P*_y_)	+*Δ*_y_/−*Δ*_y_ (*Δ*_y_)	+*P*_p_/−*P*_p_ (*P*_p_)	+*Δ*_p_/−*Δ*_p_ (*Δ*_p_)	+*P*_u_/−*P*_u_ (*P*_u_)	+*Δ*_u_/−*Δ*_u_ (*Δ*_u_)	+*μ*/−*μ* (*μ*)
PPECC-1	41.0/38.9 (40.0)	18.3/17.7 (18.0)	55.4/61.0 (58.2)	54.0/89.7 (71.9)	47.1/51.8 (49.5)	107.8/107.8 (107.8)	5.90/6.09 (6.00)
PPECC-2	35.1/36.1 (35.6)	14.5/15.4 (15.0)	51.6/61.9 (56.8)	43.5/74.6 (59.1)	43.8/52.6 (48.2)	97.2/104.5 (100.9)	6.70/6.79 (6.75)
PPECC-3	83.0/75.2 (79.1)	22.7/22.7 (22.7)	90.2/79.3 (84.8)	27.7/27.1 (27.4)	76.7/67.4 (72.1)	61.6/69.5 (65.6)	2.7/3.1 (2.9)
PPECC-4	40.7/39.9 (40.3)	18.4/17.9 (18.2)	52.4/74.0 (63.2)	44.0/52.5 (48.3)	44.5/62.9 (53.7)	83.5/87.5 (85.5)	4.54/4.89 (4.72)
PPECC-5	40.9/41.3 (41.1)	18.5/18.5 (18.5)	54.7/65.5 (60.1)	52.9/73.9 (63.4)	46.5/55.7 (51.1)	101.4/97.5 (99.5)	5.48/5.27 (5.38)
PPECC-6	38.3/37.9 (38.1)	18.0/22.0 (20.0)	56.0/51.5 (53.4)	58.3/79.8 (69.1)	47.6/43.8 (45.7)	99.8/99.7 (99.8)	5.54/4.53 (5.04)
RC-7	39.9/40.0 (40.0)	14.3/18.3 (16.3)	57.1/61.2 (59.2)	61.5/47.4 (54.5)	48.5/52.0 (50.3)	81.0/80.1 (80.6)	5.66/4.38 (5.02)
RC-8	56.0/56.3 (56.2)	24.2/21.7 (23.0)	63.9/69.2 (66.6)	59.8/41.2 (50.5)	54.3/58.9 (56.6)	60.5/60.6 (60.6)	2.50/2.79 (2.65)

Notes: *Δ*_y_ = yield displacement; *Δ*_p_ = peak displacement; *Δ*_u_ = ultimate displacement; *P*_y_ = yield load corresponding to *Δ*_y_; *P*_p_ = peak load corresponding to *Δ*_p_; *P*_u_ = ultimate load corresponding to *Δ*_u_; *μ* = *Δ*_u_/*Δ*_y_ displacement ductility factor; the superscript “+” and “−” represent the positive and negative direction, respectively; the values in parentheses are the average of the positive and negative directions.
